# Mycosis Fungoides: A Necessary Differential Diagnosis in Infectious Disease and Dermatology Settings

**DOI:** 10.1590/0037-8682-0622-2023

**Published:** 2024-03-25

**Authors:** Claudio José dos Santos, Aryanna Kelly Pinheiro Souza, Thiago José Matos Rocha

**Affiliations:** 1 Universidade de São Paulo, Faculdade de Saúde Pública, Programa de Pós-graduação em Saúde Pública, São Paulo, SP, Brasil.; 2 Universidade Estadual de Ciências da Saúde de Alagoas, Centro de Patologia e Medicina Laboratorial, Maceió, AL, Brasil.; 3 Universidade Estadual de Ciências da Saúde de Alagoas, Centro de Ciências Integradoras, Maceió, AL, Brasil.

A 69-year-old male presented to the Infectious Disease Service with erythematous scaly lesions persisting for five years, which had evolved into diffuse exfoliative erythroderma and multiple disseminated scaly plaques in the three weeks preceding admission. Physical examination revealed infiltrated facial appearance, serous blistering, ulcerated lesions in the oral and genital mucosa, infiltrated plaques on the face and auricular pavilion, crusted scaly plaques on the anterior/posterior trunk and upper and lower limbs with lesion exulceration, intense pruritus, fever, arthralgia, and diffuse lymphadenopathy w(Figure 1). Laboratory findings at admission included LDH 672 U/L, PCR 152.91 mg/dL, 75,000 leukocytes/µL, atypical lymphocytes, and convoluted nucleus lymphomatous cells suggestive of Sézary cells (10-15% in peripheral blood smears) (Figure 2). The clinical course was unfavorable, with a worsening state after 10 days, preceding specific interventions.

**FIGURE 1A AND 1B:**
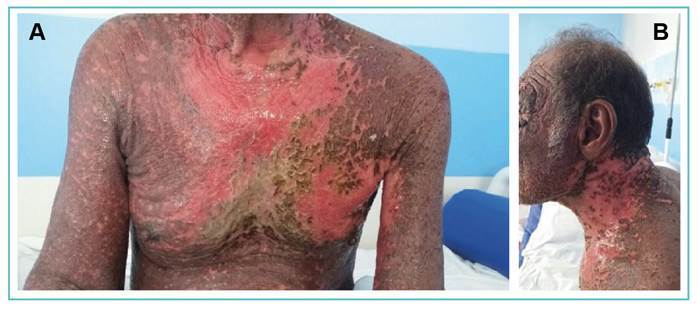
Disseminated ulcerations and erythroderma on the chest, back, and cervical regions.

**FIGURE 2A AND 2B:**
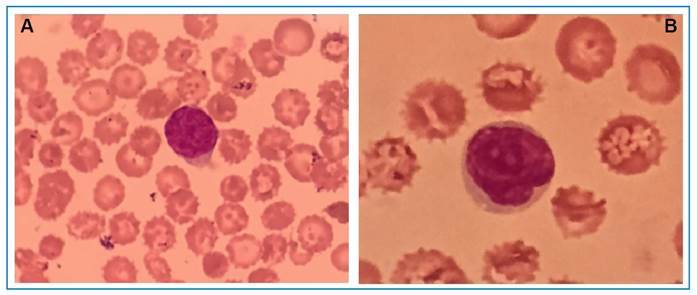
Sézary cells with convoluted nuclei in peripheral blood (Giemsa, x1000).

The challenging diagnosis of cutaneous T-cell lymphoma, particularly mycosis fungoides (MF), stems from nonspecific clinical-laboratory findings^1^. The annual incidence of T-cell cutaneous lymphoma is extremely low, at 0.77/100,000 individuals, with an estimated incidence of 0.41/100,000 for MF^2^.

In this case, the variable symptomatic manifestations did not indicate early MF. Extensive desquamative plaques and ulcerated/infected lesions were the reasons for admission, while the detection of Sézary allowed formulation of a diagnostic hypothesis.

MF significantly resembles various benign inflammatory skin conditions^3^. However, delayed diagnosis, as seen here, amplifies the likelihood of unfavorable outcomes^4^. 

Thus, a cautious approach is warranted in infectious diseases and dermatology settings, considering MF as a differential diagnosis in presentations hinting at it.

## ETHICS

The study was approved by the Institutional Ethics Committee (CAAE 33818720.6.0000.5011).

## References

[1] Amorim GM, Quintella DC, Niemeyer-Corbellini JP, Ferreira LC, Ramos-e-Silva M, Cuzzi T (2020). Validation of an algorithm based on clinical, histopathological and immunohistochemical data for the diagnosis of early-stage mycosis fungoides. An Bras Dermatol.

[2] Lobato BADL, Brito JAGSM, Carneiro TX, Xavier MB (2021). Diagnóstico tardio de micose fungoide: um relato de caso. Rev Pan-Amaz Saude.

[3] Miyashiro D, Sanches JA (2023). Mycosis fungoides and Sézary syndrome: clinical presentation, diagnosis, staging, and therapeutic management. Front Oncol.

[4] Eklund Y, Aronsson A, Schmidtchen A, Relander T (2016). Mycosis Fungoides: A Retrospective Study of 44 Swedish Cases. Acta Derm Venereol.

